# Serious Gaming for Chemotherapy-Induced Nausea and Vomiting in Older Adults With Cancer: Protocol for a Randomized Clinical Trial

**DOI:** 10.2196/64673

**Published:** 2024-10-02

**Authors:** Victoria Loerzel, Arsham Alamian, John Clochesy, Patricia I Geddie

**Affiliations:** 1 College of Nursing University of Central Florida Orlando, FL United States; 2 School of Nursing University of Miami Miami, FL United States; 3 Department of Nursing Research Moffitt Cancer Center Tampa, FL United States

**Keywords:** chemotherapy-induced nausea and vomiting, aged, serious game, symptom self-management, mobile phone, neoplasms, self-care

## Abstract

**Background:**

Older adults are at high risk for toxicity due to cancer treatment and increased risk for adverse events related to chemotherapy-induced nausea and vomiting (CINV). Unfortunately, older adults report multiple treatment-related symptoms but use few strategies to self-manage these symptoms due to erroneous beliefs related to the effectiveness of commonly taught self-management strategies. We developed a novel serious game, Managing at Home (MAH), to help older adults learn how to effectively self-manage CINV at home.

**Objective:**

This study has 2 aims. Aim 1 is to examine changes in CINV severity, self-management behaviors, functioning, quality of life, cognitive representation, and health care use within the intervention group from baseline (T_1_) to completion of the study (T_6_). Aim 2 is to determine the efficacy of the MAH intervention by comparing differences in primary outcomes (CINV severity and health care use) and secondary outcomes (self-management behaviors, functioning, and quality of life) between the intervention and control groups at each follow-up visit (T_2_-T_6_) and completion of the study (T_6_).

**Methods:**

This is a longitudinal randomized clinical trial. We will collect data from 500 older adults receiving cancer-related chemotherapy at baseline (T1) and at each treatment cycle until cycle 6 (T6). Participants will be enrolled if they are 60 years or older of age, are newly diagnosed with cancer, being treated with any chemotherapy agent with moderate or high emetic potential, are on a 2-, 3-, or 4-week treatment cycle, are proficient in English, and have a telephone. Previous diagnosis or treatment for cancer, end-stage disease with less than 6 months to live, and uncorrected visual or hearing impairment are exclusion criteria.

**Results:**

This study was funded in September 2022 and received institutional review board approval in October 2022. As of July 2023, the enrollment of participants is ongoing and currently has 130 enrolled participants. Data collection and analysis will be complete in 2027.

**Conclusions:**

This study addresses self-management of CINV in older adults using an innovative serious game. The MAH intervention uses simulation and gaming technology to engage older adults in active learning in order to reframe erroneous perceptions about symptom self-management. If shown to be effective, it can easily be adapted to include other cancer-related symptoms or other chronic illnesses.

**Trial Registration:**

ClinicalTrials.gov NCT05838638; https://clinicaltrials.gov/study/NCT05838638

**International Registered Report Identifier (IRRID):**

DERR1-10.2196/64673

## Introduction

### Background

Older adults (≥65 years) represent about 15% (46 million) of the US population, and the rate is expected to double (90 million) by 2050 [1-3]. They also represent 55% of all cancer diagnoses [2,4]. With an aging population, the number of older adults with cancer will also increase, taxing limited health care resources. Despite advances in antiemetic therapy, older adults diagnosed with cancer experience chemotherapy-induced nausea and vomiting (CINV) [5]. Although older adults are less likely to have CINV symptoms, they are more likely to experience serious consequences (eg, dehydration and renal impairment) than younger adults [6-8] due to the presence of multiple comorbidities, compromised organ function, frailty, and decreased overall functioning [9-11]. CINV occurs in up to 80% of adults receiving chemotherapy [12,13] and can lead to metabolic and nutritional issues (eg, loss of appetite, weight loss, and malnutrition), decrease in self-care and functioning, decline in physical and mental status, and poor quality of life (QOL) [12]. Up to 47% of adults receiving moderate to high emetogenic chemotherapy will experience a hospital admission (HA) related to CINV [14,15] and ~20% will have an unplanned emergency department (ED) visit [16]. The Centers for Medicare and Medicaid Services [17] urge improving care and reducing unplanned ED visits in those receiving cancer treatment. Therefore, interventions are needed to decrease CINV symptoms, improve functioning and QOL among older adults, and reduce health care use due to poor CINV symptom self-management.

Despite clinical guidelines for managing CINV, ~49% of adults with cancer are undertreated for CINV [13,18], and prevention of CINV remains a challenge [5]. Risk factors for undertreatment include the type of chemotherapy, being treated in the outpatient clinic, and out-of-pocket costs for antiemetics [18]. Health care practitioners report that managing breakthrough nausea within the first 5 days after chemotherapy is a common occurrence, while managing delayed CINV is the most challenging [19]. About 32% of practitioners report the need to stop or delay cancer treatment in patients due to CINV [19].

While the above risk factors for CINV are beyond the scope of this study and control of patients with cancer, there are many pharmacologic and nonpharmacologic strategies that older adults can use to prevent or self-manage CINV and minimize negative effects. Older adults diagnosed with cancer experience multiple side effects from chemotherapy treatment, yet do very little to manage those side effects [20,21]. In general, older adults modify their lives to reduce the severity of side effects rather than engaging in active self-management of those side effects [22-25]. Older adults are slower to take action against symptoms than younger adults [26], tend to minimize symptoms and their consequences [27-29], and normalize and interpret symptoms as part of aging, leading them to delay seeking or starting treatment [26,30]. They also delay treatment due to being uncertain about the symptom’s severity or seriousness and believing that treatment or management is not possible [28]. Some older adults believe that side effects have to be endured as part of their cancer treatment [31,32]. However, those who believe they can manage their symptoms engage in self-management strategies [33]. Interventions, such as the Managing at Home (MAH)-CINV [34], which specifically address older adults’ erroneous symptom beliefs and encourage them to engage in preventative and self-management behaviors may be able to alter their responses to side effects due to cancer treatment [35].

Printed standard patient education material for CINV based on well-known treatment and prevention guidelines (Textbox 1) are routinely provided to new patients and their family members so they can self-manage CINV at home. However, there is very little research related to how older adults self-manage side effects at home during chemotherapy treatment. Prior research by this team shows that without additional educational intervention, only ~25% of older adults who experience nausea actively manage it with medication [21]. Instead, they prefer to modify their activities or use dietary strategies to manage their nausea [21]. However, we found that with additional technology-based education and intervention and a change to their erroneous symptom beliefs, preventative and self-management strategies improve overall with twice as many older adults engaging in CINV prevention activities compared to a control group [34]. Results from our pilot study using the MAH educational intervention indicate that over 55% of participants used hydration as a strategy to prevent CINV [34]. Dietary strategies, taking antiemetics, and relaxation techniques were the top appropriate preventative and self-management strategies used by participants [34]. Although our educational intervention increased the use of these strategies, our sample size was too small to examine the effectiveness of the MAH intervention to improve the CINV symptoms*.* Therefore, the goal of this project is to examine the effectiveness of the MAH intervention in reducing CINV symptoms among older adults.

At the time that our team developed the MAH intervention, few studies used technology-based platforms to educate older adults. Those who used these platforms focused on Wii Fit games to promote exercise [36], mobile devices to increase physical activity [37], and avatars and touchscreen technology to manage diabetes [38]. However, since then, serious gaming interventions are becoming more popular. Serious gaming is a well-established method designed to support learning and change health-related behavior by engaging individuals in an entertaining experience [39-41]. Serious gaming capitalizes on fun while promoting behavior change [40]. It is also consistent with our goal of changing behavior by changing their beliefs about self-care related to CINV.

Recent research has used serious gaming among older adults to increase self-management knowledge about heart failure [42], detect cognitive impairment [43], provide cognitive task training [44], promote active lifestyle [45], provide education about pain management after surgery [46], detect frailty through grip strength [47], and provide training on how to avoid falls [48]. All of these studies have shown that serious gaming is usable, feasible, and acceptable for use in older adults. Our pilot study showed that it is feasible to recruit older adults diagnosed with cancer to participate in our intervention using serious gaming, and the MAH has high acceptability among users [34]. Coupled with the positive impact the MAH had on increasing preventative and self-management behaviors, this novel intervention shows promise in influencing how older adults think about CINV management and engage in self-management behaviors.

Older adults are using more technology on a day-to-day basis and becoming more proficient. A 2020 American Association of Retired Persons report states that older adults are embracing new technology, with over 50% purchasing some sort of technological product in the past year. Over half of the population aged 50 years and older own a smart tablet, and about 77% of older adults own a smartphone [49]. Another 2021 American Association of Retired Persons report states that older adults increased their use of technology during the recent COVID-19 pandemic and have bridged the “digital gap” between them and younger generations. Due to the pandemic and resulting social isolation, older adults, including people aged 70 years and older purchased, and used, more devices such as smartphones, tablets, and wearables [50]. This shows that older adults are embracing technology and are becoming more literate with the technology. This supports the use of our technology-based intervention and data collection strategies.

**Common recommended chemotherapy-induced nausea and vomiting interventions.**

**Pharmacologic**

- Medication
  Antiemetic therapy as prescribed by the physician

**Nonpharmacologic**

- Nutrition
  Eat dry food, small meals, and bland and easy to digest foods
  Sit up after eating
  Avoid cooking odors
  Avoid spicy and greasy foods
- Hydration
  Drink 8 to 12 cups of liquid per day
  Drink even if not thirsty
  Include liquids like soup, ice pops, flavored ices, and gelatin
  Limit caffeine products
- Complementary
  Relaxation, yoga, behavioral therapy (distraction and meditation), guided imagery, controlled breathing, environmental changes, and acupuncture or acupressure

### Aims and Hypotheses

The goal of this project is to examine the effectiveness of the MAH intervention in reducing CINV severity and health care use and increasing functioning and QOL. We aim to:

- Aim 1: Examine changes in CINV severity, self-management behaviors, functioning, QOL, cognitive representation, and health care use within the intervention group (IG) from baseline (T_1_) to completion of the study (T_6_).
  We hypothesize that the IG will report lower levels of CINV severity and health care use and higher levels of self-management, functioning, cognitive change, and QOL from baseline to the completion of the study.
- Aim 2: Determine efficacy of the MAH intervention by comparing differences in primary outcomes (CINV severity and health care use) and secondary outcomes (self-management behaviors, functioning, and QOL) between the IG and control group at each follow-up visit (T_2_-T_6_) and completion of the study (T_6_).
  We hypothesize that the IG will report lower levels of CINV severity and health care use at T_2_-T_6_ than the control group.
  We hypothesize that the IG will report higher levels of self-management behaviors, functioning, and QOL at T_2_-T_6_ than the control group.

## Methods

### Study Design

Using a randomized clinical trial, we will enroll older adults from 2 clinical sites in Florida. Participants will be randomized to either the IG or attention control group (ACG). The IG will receive the MAH at baseline, and the ACG will receive it at the end of the study. Data will be collected over 6 months or 6 chemo cycles (T_2_-T_6_). Data related to CINV severity, self-management, functioning, QOL, and health care use at baseline and at the 6 time points will be collected from both groups.

### Settings

The intervention will be offered at outpatient treatment centers at a community cancer center in Orlando, Florida, and a Comprehensive Cancer Center in Miami, Florida, that provide care to over 6000 adults aged 60 years and older each year. These numbers indicate an ample sampling pool of older adults diagnosed with cancer.

### Study Participants

A sample of 500 participants will be enrolled if they meet the following inclusion criteria: ≥60 years of age, newly diagnosed with cancer, treatment with any chemotherapeutic agent of moderate or high emetic potential [51], on a 2-, 3- or 4-week treatment cycle, proficient in English, and have a telephone. Exclusion criteria are previous diagnosis or treatment for cancer, end-stage disease with less than 6 months to live, and visually or hearing impaired without corrective device. Dementia or cognitive impairment is not a concern with this population since they have already been cognitively screened to consent to treatment at each site. These criteria were also successfully used in our pilot study.

### Power Analysis and Sample Size

For aim 1, when the outcome of interest is continuous (eg, CINV severity and number of self-management behaviors), a sample of 100 participants, measured continuously throughout the study period between T1 and T6, achieves a power of 0.904 when using a chi-square test from a generalized estimating equation (GEE) analysis to test whether the average slope of the participants differs significantly at a significance level of .05. The residual SD is anticipated to be 1.00. Missing values are assumed to occur completely at random (missing completely at random [MCAR]). These missing value proportions will be combined to form the pairwise observant probabilities using the independent method. The anticipated proportions missing at each measurement time are 0.00, 0.08, 0.16, 0.24, 0.32, and 0.40. The first row of the autocorrelation matrix of the responses within a participant is assumed to be 1.0000, 0.4000, 0.1600, 0.0640, 0.0256, and 0.0102. Other rows follow the same pattern. For aim 1, a total of 254 participants, each scheduled to be measured 6 times, achieves a power of 0.90 when using a 2-sided Wald test from a GEE analysis with a count outcome (eg, QOL) to test whether a μ1(1) of 0.500 differs from a μ2(1) of 1.000 at a significance level of .05. μ1(0) and μ2(0) are assumed to be 1.000. Missing values are assumed to occur completely at random (MCAR). These missing value proportions will be combined to form the pairwise observant probabilities using the independent method. The anticipated proportions missing at each measurement time are 0.00, 0.08, 0.16, 0.24, 0.32, and 0.40. The first row of the autocorrelation matrix of the responses within a participant is assumed to be 1.000, 0.500, 0.250, 0.125, 0.063, and 0.031. Other rows follow the same pattern.

For aim 1, when the outcome of interest in binomial (eg, resource use), a sample of 357 participants, each scheduled to be measured 6 times, achieves a power of 0.90 when using a 2-sided Wald test from a GEE analysis with the binary outcome to test whether a P1(1) of 0.500 differs from a P2(1) of 0.250 at a significance level of .05. P1(0) and P2(0) are assumed to be 0.500. Missing value (MCARs) proportions will be combined to form the pairwise observant probabilities using the independent method. The anticipated proportions missing at each measurement time are 0.00, 0.08, 0.16, 0.24, 0.32, and 0.40. The first row of the autocorrelation matrix of the responses within a participant is assumed to be 1.000, 0.500, 0.250, 0.125, 0.063, and 0.031. Other rows follow the same pattern.

For aim 2, based on the assumption of a nausea severity mean score of 6.20, range from 2 to 9, an SD of 2.44, and a clinically meaningful difference in mean nausea severity score of 1 between the IG and control group, a sample size of 500 participants (250 per group), measured between 6 and up to 30 times (depending on the outcome variable of interest), achieves 87% power to detect a difference between the (fixed) group means. The ratio of the subject-specific slope variance to σ² is 0.100. The correlation between measurements within a participant is 0.100. The test was based on a mixed model regression analysis using a significance level of .05. This power analysis was calculated using PASS 20 (NCSS LLC). Anticipating an attrition rate of ~22%, we will seek to enroll 610 older adults for this study. Attrition to mortality is not expected to be high due to the exclusion criteria for those with less than 6 months to live.

### Recruitment, Randomization, and Procedures

Successful recruitment and retention strategies previously established by our team will guide this study [27,34,52-54]. A stratified sampling strategy followed by randomization will be used. This sample will mirror Florida demographics and aim to recruit participants in the following percentages: female (51%), male (49%), Black (16%), Hispanic (26.4%), White (77%), and other races (7%). At both clinical sites, a research assistant will screen all new patients for eligibility using their respective electronic medical record systems. Eligible patients will be approached by the research assistant at their first chemotherapy appointment and asked to participate. In our pilot study, this timing acted as a distraction for patients while they waited for chemotherapy to start and did not interfere with treatment, nursing care, or education. In addition, prior to treatment, all patients have received standard written education regarding treatment effects and will expect CINV as a possible effect of treatment; therefore, being approached about the study is not expected to elicit anxiety. Informed consent will be obtained in a private area of the clinic open to the research assistant, and a copy of the consent form will be given to the participant.

Once informed consent is obtained, baseline measures will be completed, and participants will be randomized (IG and ACG) to either group by choosing an envelope with a group assignment. If they are randomized to the IG, the intervention will begin immediately. Regardless, participants in each group will receive standard care (1) on admission to the cancer center (receipt of standard written educational materials that patients are instructed to bring to each appointment), (2) at a treatment planning visit in the clinic (receipt of specific handouts regarding treatment and potential side effects, and (3) at time of treatment (verbal review of chemotherapy and symptom handouts by treatment center or chemotherapy nurses). Retention of all participants will be enhanced with (1) weekly follow-up telephone calls and (2) ACG offered MAH at the end of the study.

### Intervention

The intervention consists of three parts: (1) the MAH serious game, (2) self-care discussion with the research assistant, and (3) follow-up.

#### Part 1: The MAH Serious Game

The game will be administered and supervised by the research assistant. Participants will play the game on an iPad at the outpatient treatment center prior to their first chemotherapy treatment. Headphones can be used to ensure privacy and limit distraction. During the game, players make decisions for the avatar based on common CINV management practices (eg, taking medication, dietary strategies, and staying hydrated; Textbox 1), which cause the avatar to become nauseated or be nausea-free.

#### Part 2: Self-Care Discussion With the Research Assistant

At the end of the game, the research assistant will answer any questions the participant has and make clarification when needed. In the event, if a participant’s avatar has a negative outcome (ie, severe nausea), self-management behaviors will be corrected by the research assistant. In our pilot study, these discussions varied between none and 1 or 2 questions from participants. Based on our pilot data, we estimate that 10% (n=25) of players needed a more in-depth discussion to correct self-management behaviors. In this study, we will document the types of questions asked and the content of these discussions to improve the internal validity of the intervention. We will also document the involvement of caregivers in the intervention, if it occurs.

#### Part 3: Follow-Up

Participants will be allowed to play the game as many times as they choose during that chemotherapy visit or at subsequent visits in order to practice making decisions and visualizing consequences and outcomes. Once the participant is discharged home, the graduate research assistant (GRA), blinded to the treatment group, will call participants weekly to see how they are doing and remind them to respond to data collection texts or use paper or pencil forms. To reduce bias, a scripted interview form will be used, and no “teaching” will occur. Calls will only be to check in with the participants and remind them to collect data. If participants ask questions about managing CINV and other symptoms, the GRA will instruct them to contact their oncology team for additional management. In addition, participants will be asked to follow cancer center procedures and call their oncologist if they are having problems or have questions.

### Treatment Fidelity and Monitoring

The MAH will be presented and delivered by research assistants who have been trained in the game and CINV symptom management strategies. The research assistant will answer questions during the decision discussion based on common self-management options (Textbox 1). A checklist for each interaction with the participant will guide each encounter. Minor interruptions (<10 minutes) to gameplay will be recorded, and participants will resume gameplay where they left off. Technology malfunctions (eg, frozen screen) will be recorded, and participants will be asked to start MAH over again using a new iPad.

### End Points

The study will continue until one of the following end points is met: (1) completion of the study, (2) no longer receiving chemotherapy treatment, or (3) unreasonable delays in treatment.

### Control Group

Participants in the control group will receive (1) standard care, (2) follow-up phone calls from the GRA to remind them to reply to data collection texts, and (3) the opportunity to play MAH at the end of the study. To reduce bias, a scripted interview form will be used, and no “teaching” will occur. Calls will only be to check in with the participants and remind them to collect data. If participants ask questions about managing CINV and other symptoms, the GRA will instruct them to contact their clinic nurse for additional management. In addition, participants will be asked to follow cancer center procedures and call their oncologist if they are having problems or have questions. Contamination between groups is not expected since participants are likely scheduled for chemotherapy at different times and receiving treatment in different areas of the treatment center.

### Outcome Measures

#### Primary Outcome Measures

##### Health Care Resource Use

Health care resource use is defined as any unplanned ED admission or HA and will be recorded via participant report using a dichotomous(Y/N) variable at each follow-up phone contact, text, and treatment visit. Follow-up questions about the reason for the admission, length of time for admission, and treatment will also be asked. The report will be verified by medical record review whenever possible.

##### CINV Severity

CINV severity will be collected on the web using texted links to surveys and will include the presence and severity of CINV (0-10 Likert scale). Participants without a smartphone will be provided the paper and pencil Symptom Management Checklist (SMC; see Data Collection section) to record this data.

#### Secondary Outcome Measures

##### Self-Management Behaviors

CINV prevention and self-management actions, effectiveness of actions (0-10 Likert scale), and amount of hydration will be recorded using the SMC that was used in the pilot study to measure CINV severity and prevention and self-management behaviors. Participants will be asked to record their highest level of nausea each day and preventative self-management actions for CINV and hydration. When experiencing CINV, the participant will use the SMC to record (1) CINV severity (0-10 Likert scale) prior to self-management, (2) the type of self-management strategies used, and (3) the strategies perceived helpfulness in reducing nausea (0-10 Likert scale). All self-management strategies are coded (eg, “MED”=taking antiemetic and “DIS”=using distraction), and the participants will fill in the strategy code they are using on the form. Participants with a smartphone will complete this form electronically, and those without a smartphone will use the paper or pen version, which will be collected by the research assistant at each chemotherapy cycle visit.

##### Symptom Severity

Part I of the Symptom Representation Questionnaire [55] has a 24-item self-report measure that will be used to identify the presence and severity of common treatment-related symptoms (0- to 10-point Likert scale), including nausea and vomiting. This measure asks respondents to think about their symptoms over the past week.

##### Cognitive Representation

Part II of the Symptom Representation Questionnaire [55] has an additional 15 questions to assess cognitive representation (identity, cause, timeline, consequences, and cure or control) or beliefs about specific symptoms (0-4 agreement scale). This measure includes subscales for symptom cause (2 questions), timeline (3 questions), cure or control (3 questions), and consequence (6 questions). Only the cognitive representation of nausea will be evaluated. Original reliability statistics for this instrument were fair (Cronbach α=0.63-0.88 for the subscales) [55], and our pilot showed a Cronbach α of 0.64. The cognitive representation questions will be modified at baseline to assess participants’ beliefs about potential CINV consequences and their ability to be controlled or cured. These data will be collected again at the end of the study.

##### Health-Related QOL

The European Organization for Research and Treatment of Cancer-30 [56] will measure QOL at each treatment cycle. This frequency is consistent with other studies that have measured QOL in older adults over time [57,58]. This well-known scale includes 5 functional scales, 3 symptom scales, and 6 single items using a 4-point Likert scale (1=not at all to 4=very much), and 2 global health or QOL items using a 7-point Likert scale (1=very poor to 7=excellent). Higher scores on the functional and health or QOL scale indicate better functioning, while higher scores on the symptom-related scales and items indicate higher symptoms. The tool was previously shown to be reliable in studies with older adults (Cronbach α=0.89) [56] and had a Cronbach α of 0.80 in our pilot study.

#### Additional Measures

##### Sociodemographic Characteristics

Sociodemographic characteristics include age, gender, race or ethnicity, comorbidity information, education level, and income level. These characteristics will be measured at T_1_ (baseline).

##### Experiential Characteristics

Experiential characteristics include cancer diagnosis and stage, chemotherapy regimen with antiemetics, additional treatments (eg, radiation therapy), discharge antiemetic prescriptions, fluids during treatment, changes to the treatment plan (ie, delays in treatment, chemotherapy dose reductions, and changes in chemotherapy), and number of times game is played. All cancer information will be collected from the patient’s electronic medical record by the research assistant.

### Data Collection

Once enrolled in the study (T_1_—baseline), research assistants will electronically record information about the participants’ diagnosis and treatment plan collected from medical records. Participants in both groups will complete baseline questionnaires electronically on an iPad given to them by the research assistant. The research assistant will demonstrate how to complete the electronic questionnaire and assist with any questions or issues that may occur during data collection. The research assistant will also meet with participants at subsequent treatment cycles (T_2_-T_6_) to have them fill out the electronic questionnaires on the iPad. Assistance will be given as needed.

Between treatment cycles, we will collect information about CINV severity, self-management behaviors, and any ED visit or HA from all participants. Participants with smartphones will be asked to respond to SMS text messages that will provide a link to the SMC, where they will electronically record information about their CINV severity, self-management behaviors, and any ED visit or HA. Participants will receive SMS text messages daily for the first week after treatment and then every 3 days until their next cycle of chemotherapy. This pattern will repeat at each treatment cycle. Current research supports the use of SMS text messaging since it is estimated that ~77% of older adults use smartphones. In total, 81% of adults ages 60 to 69 years report use of smartphones [49], and 79% of those ages 70-74 years report use of smartphones [59]. Research assistants will provide a tutorial on how to use the electronic links and enter data prior to the end of the first appointment. Participants without smartphones will be given a paper and pencil version of the SMC. They will be asked to record data on their daily CINV severity, self-management behaviors, and any ED visit or HA. The paper and pencil method of data collection was successfully used in the pilot study with ~75% of participants at least tracking fluids and ~64% tracking self-management behaviors over time [34]. All participants will be contacted weekly by a GRA, blinded to their treatment group, to assess how they are doing, answer any study-related questions, and encourage participants to reply to the texts or complete the paper and pencil data collection. The GRA will not manage the care of participants while they are in the study, instead, they will refer participants to their oncology team if they have questions or concerns about treatment-related side effects they are experiencing.

### Data Management and Integrity

All data will be stored using REDCap (Research Electronic Data Capture; Vanderbilt University) [60], a secure web application database, which will be accessed by a secure web connection with authentication and data logging. Due to the multisite feature of this study, 2 separate REDCap systems will be set up on the University of Miami and University of Central Florida servers separately, but they will share the same structure so that the data could be communicated and analyzed in a consistent way. For surveys that will be filled out by participants electronically, a secured and unique link will be sent via email or text so that the data can be captured accurately. A unique identifier associated with each participant will also be incorporated into the surveys so that the survey responses could be easily identified and rechecked by research staff. Research assistants will also use this identifier when they enter data from the electronic medical record and from their checklists. For any paper and pen surveys, the project director or research assistants will review all data for completeness and appropriate entries before it is entered into the study database.

At the beginning of the data entry process, a minimum of 1 in every 10 participant records will be checked against the original record for data errors. Records for review will be randomly chosen by the data manager. As the study progresses, the frequency of monitoring will be based on the results of the previous data review. If the error rate is unacceptable, then the monitoring will increase until the error rate is acceptable (less than 5% of all data). In consultation with the principal investigator (VL), the data manager will develop reports that will help in the coordination and management of the project and also allow project staff to monitor the quality of the data and the progress of the study. The data files will be backed up daily during the data entry process and once a month when data collection is complete by the data manager. All data files will be housed on the university servers, which are backed up every 24 hours, and copies will be stored off-site for additional security. Access to the database will be password-protected and limited to the investigators, research personnel, project director, and data manager. Furthermore, the user rights will be assigned appropriately by the project manager or data manager to ensure data security and privacy.

### Data Analysis

#### Overview

Prior to the testing of hypotheses and modeling of outcome data, a detailed descriptive analysis of all data collected will be performed, involving the summarization of data and the use of exploratory data analytic techniques. This preliminary exploration of the data will be used to (1) verify randomization by assessing the comparability of groups on key descriptors at baseline, (2) describe univariate and bivariate sample distributions of the data, (3) ascertain the interrelationships between variables, and (4) check for the violation of assumptions necessary for parametric statistical techniques. Specifically, appropriate statistics describing the location (eg, means and percentiles) and dispersion (eg, SDs and ranges) of the distributions of each continuous type variable will be computed for the total sample and over time (when applicable). Frequency counts and percentages will be calculated for categorical variables. Normality will be assessed graphically (eg, histograms and normal probability plots) and inferentially (eg, skewness and kurtosis divided by their SE and Shapiro and Wilk *W* statistic). Levene test for equality of variances will be used to test the assumptions for all analyses using parametric statistics to compare means. If the necessary statistical assumptions are severely violated whereby compromising the validity of the proposed statistical procedure, data transformations or alternate statistically robust approaches, such as analogous nonparametric approaches, will be used.

#### Aim 1

Aim 1 examines changes in CINV severity, self-management behaviors, functioning, QOL, cognitive representation, and health care use within the IG from baseline (T_1_) to completion of the study (T_6_). We hypothesize that the IG will report lower levels of CINV severity and health care use and higher levels of self-management, functioning, cognitive change, and QOL from baseline to the completion of the study.

Changes in primary and secondary outcomes from T1 to T6 will be assessed using linear (CINV severity, self-management behaviors, cognitive representation, and functioning), binary (resource use), and ordinal (QOL) regressions, as appropriate, within a GEE framework. All models will control for the effects of 7 demographic (age, gender, race or ethnicity, comorbidity, education level, income level, and insurance status) and 5 experiential (cancer type, emetic potential, treatment frequency, intake of IV fluids between treatment cycles, and number of home antiemetics) variables. GEE models are well suited for this type of repeated measures design, as they account for within-subject correlations between repeated measurements over time in a longitudinal design. Furthermore, GEE models are not restricted by any distributional assumptions including the assumptions of normality required by most other approaches to longitudinal analysis such as repeated measures ANOVA. Finally, GEE models provide robust parameter and SE estimates even if the working correlation structure for the data is misspaced [61]. The analysis will be 2-tailed and conducted with SAS (version 9.4; SAS Institute Inc).

#### Aim 2

Aim 2 determines the efficacy of the MAH intervention by comparing differences in primary outcomes (CINV severity and health care use) and secondary outcomes (self-management behaviors, functioning, and QOL) between the IG and control group at each follow-up visit (T_2_-T_6_) and completion of the study (T_6_). We hypothesize that the IG will report lower levels of CINV severity and health care use at T_2_-T_6_ than the control group. We hypothesize that the IG will report higher levels of self-management behaviors, functioning, and QOL at T_2_-T_6_ than the control group.

The effect of the intervention on primary and secondary outcomes at each follow-up visit and at the completion of the study will be assessed using generalized linear mixed models (GLMMs). GLMMs are an extension of general linear models allowing the study of within- and between-subjects repeated measures variations over time. GLMMs can also be used to fit normal and nonnormal response variables, as it is the case in this study. The interaction of time and treatment will be assessed by using an interaction term in the longitudinal models. All models will control for the effects of demographic and experiential variables listed in aim 1. The analysis will be 2-tailed and conducted with SAS (version 9.4).

### Ethical Considerations

This study was approved by the institutional review board (IRB) at the University of Central Florida on October 7, 2022 (STUDY00003948). This IRB also serves as a single IRB. Written informed consent will be obtained prior to any data collection and randomization. Study data will be deidentified and stored on password-protected servers that will be accessible only to the study team. Individual participants will not be identified in any presentation or publication materials. Participants are compensated with a US $75.00 gift card at the end of their participation in the study.

## Results

This study was funded in September 2022 and received IRB approval in October 2022. As of July 2023, the enrollment of participants is ongoing and currently has 130 enrolled participants. Data collection and analysis will be complete in 2027.

## Discussion

### Preliminary Findings

Our MAH intervention is focused on changing older adults’ perceptions about managing CINV and helping them to learn to use more self-management strategies at home to avoid unplanned HA and ED admission. Our goal is to examine the effectiveness of the MAH intervention in reducing CINV severity and health care use while increasing functioning and QOL.

Our pilot study (n=80) showed small effect sizes and no significant difference between the IG and control group for the main outcome variables of nausea, vomiting, QOL, or health care use. However, the IG reported being more engaged in self-care behaviors to prevent nausea compared to the control group. The IG reported using twice the number of self-management strategies to prevent nausea compared to the control group (1486 vs 768), while the control group reported using twice the number of self-management strategies to manage CINV (1311 vs 681) once they had it [31]. The pilot study also demonstrated the interventions’ feasibility and also showed the game was easy to play and useful in helping them manage CINV at home [31]. These results support that the MAH intervention may have shifted prior beliefs related to the timing of symptom self-management at home and increased the belief that engaging in self-management was beneficial.

### Future Implications

This study addresses a common and serious side effect of chemotherapy that often leads to severe toxicity and unplanned HA and ED admission in older adults with cancer. It also supports using new technologies, such as serious games, as an educational tool for older adults. If effective, this program can be expanded to include scenarios for multiple symptoms that are typically experienced simultaneously by people under treatment for cancer. In addition, the intervention can be culturally adapted for multiple populations.

### Limitations, Potential Challenges, and Alternatives

#### Recruitment and Retention

Although recruitment and retention of older adults in a clinical trial may be challenging, this study will use successful procedures developed in the pilot study. Diverse recruitment will be enhanced by the addition of the Sylvester Comprehensive Cancer Center, which is located in Miami, Florida, where over 60% (approximately 276,173 people) of the population is Hispanic. Retention will be enhanced through the availability of the MAH intervention at the end of the study for the ACG and follow-up phone calls for both groups.

#### Respondent Burden

Data will be collected electronically at each time point while the participants are at the cancer centers receiving treatment and is expected to take 15-20 minutes at T_1_ and no longer than 5 minutes at subsequent visits (T_2_-T_6_). In addition, SMS text messages or daily pen or pencil forms will be used to collect data between treatment visits and will consist of only 7 questions. Texts will be reduced from every day in the first week after treatment to every third day to reduce the burden on participants.

#### Lack of Technology

Older adults who do not own a smartphone will be able to provide symptom management data in a paper or pen format, which was successful in the pilot study.

### Conclusions

Older adults are at risk for severe cancer treatment–related side effects and often neglect to engage in symptom prevention or self-management behaviors, which can lead to unplanned ED admission and HAs for treatment. Finding new and innovative ways, like the MAH intervention, to educate older adults about symptom management is critical. The significance of the proposed intervention lies in its ability to allow older adults to practice making decisions about CINV self-care, visualize the consequences of self-management decisions, and reframe their beliefs (cognitive representation) about symptom self-management. MAH is poised to reduce health care costs by decreasing the use of resources (unplanned ED admission or HAs) for CINV due to poor symptom self-management. This research aligns with the National Institute of Nursing Research [62] priority of symptom reduction through self-management and the Centers for Medicare and Medicaid Services [17] priority of improving care and reducing unplanned ED visits in those receiving cancer treatment.

## Abbreviations

ACG: attention control group
CINV: chemotherapy-induced nausea and vomiting
ED: emergency department
GEE: generalized estimating equation
GLMM: generalized linear mixed model
GRA: graduate research assistant
HA: hospital admission
IG: intervention group
IRB: institutional review board
MAH: Managing at Home
MCAR: missing completely at random
QOL: quality of life
REDCap: Research Electronic Data Capture
SMC: Symptom Management Checklist

## Data Availability

The datasets generated during this study are available from the corresponding author upon reasonable request.

## References

[1] Sharpless N (2018). The challenging landscape of cancer and aging: charting a way forward. National Cancer Institute.

[2] Mather M, Jacobsen LA, Pollard KM (2015). Aging in the United States. Popul Bull.

[3] (2020). Demographic changes and aging population. Rural Health Information Hub.

[4] Howlader N, Noone A, Krapcho M, Miller D, Brest A, Yu M (2019). SEER Cancer Statistics Review, 1975-2016. National Cancer Institute.

[5] Aapro M (2018). CINV: still troubling patients after all these years. Support Care Cancer.

[6] Koll T, Pergolotti M, Holmes HM, Pieters HC, van Londen GJ, Marcum ZA, MacKenzie AR, Steer CB (2016). Supportive care in older adults with cancer: across the continuum. Curr Oncol Rep.

[7] Nishijima TF, Deal AM, Williams GR, Sanoff HK, Nyrop KA, Muss HB (2018). Chemotherapy toxicity risk score for treatment decisions in older adults with advanced solid tumors. Oncologist.

[8] O'Neill CB, Baxi SS, Atoria CL, O'Neill JP, Henman MC, Sherman EJ, Lee NY, Pfister DG, Elkin EB (2015). Treatment-related toxicities in older adults with head and neck cancer: a population-based analysis. Cancer.

[9] Feliu J, Heredia-Soto V, Gironés R, Jiménez-Munarriz B, Saldaña J, Guillén-Ponce C, Molina-Garrido MJ (2018). Can we avoid the toxicity of chemotherapy in elderly cancer patients?. Crit Rev Oncol Hematol.

[10] Bhatt VR (2019). Cancer in older adults: understanding cause and effects of chemotherapy-related toxicities. Future Oncol.

[11] Le Saux O, Falandry C (2018). Toxicity of cancer therapies in older patients. Curr Oncol Rep.

[12] (2019). Nausea and vomiting related to cancer treatment (PDQ®)—health professional version. National Cancer Institute.

[13] Hernandez Torres C, Mazzarello S, Ng T, Dranitsaris G, Hutton B, Smith S, Munro A, Jacobs C, Clemons M (2015). Defining optimal control of chemotherapy-induced nausea and vomiting-based on patients' experience. Support Care Cancer.

[14] Numico G, Cristofano A, Mozzicafreddo A, Cursio OE, Franco P, Courthod G, Trogu A, Malossi A, Cucchi M, Sirotovà Z, Alvaro MR, Stella A, Grasso F, Spinazzé S, Silvestris N (2015). Hospital admission of cancer patients: avoidable practice or necessary care?. PLoS One.

[15] Schwartzberg L, Harrow B, Lal LS, Radtchenko J, Lyman GH (2015). Resource utilization for chemotherapy-induced nausea and vomiting events in patients with solid tumors treated with antiemetic regimens. Am Health Drug Benefits.

[16] Loerzel VW, Hines R, Deatrick CW, Geddie PI, Clochesy JM (2021). Unplanned emergency department visits and hospital admissions of older adults under treatment for cancer in the ambulatory/community setting. Support Care Cancer.

[17] (2016). CMS finalizes hospital outpatient prospective payment changes for 2017. Centers for Medicare & Medicaid Services.

[18] Mahendraratnam N, Farley JF, Basch E, Proctor A, Wheeler SB, Dusetzina SB (2019). Characterizing and assessing antiemetic underuse in patients initiating highly emetogenic chemotherapy. Support Care Cancer.

[19] Van Laar ES, Desai JM, Jatoi A (2015). Professional educational needs for chemotherapy-induced nausea and vomiting (CINV): multinational survey results from 2388 health care providers. Support Care Cancer.

[20] Loerzel VW (2015). Symptom experience in older adults undergoing treatment for cancer. Oncol Nurs Forum.

[21] Loerzel VW (2018). Symptom self-management: strategies used by older adults receiving treatment for cancer. Clin J Oncol Nurs.

[22] Closs SJ, Staples V, Reid I, Bennett MI, Briggs M (2007). Managing the symptoms of neuropathic pain: an exploration of patients' experiences. J Pain Symptom Manage.

[23] Fitch MI, Mings D, Lee A (2008). Exploring patient experiences and self-initiated strategies for living with cancer-related fatigue. Can Oncol Nurs J.

[24] Speck RM, DeMichele A, Farrar JT, Hennessy S, Mao JJ, Stineman MG, Barg FK (2012). Scope of symptoms and self-management strategies for chemotherapy-induced peripheral neuropathy in breast cancer patients. Support Care Cancer.

[25] Spichiger E, Rieder E, Müller-Fröhlich C, Kesselring A (2012). Fatigue in patients undergoing chemotherapy, their self-care and the role of health professionals: a qualitative study. Eur J Oncol Nurs.

[26] Leventhal EA, Prohaska TR (1986). Age, symptom interpretation, and health behavior. J Am Geriatr Soc.

[27] Loerzel VW, Aroian K (2012). Posttreatment concerns of older women with early-stage breast cancer. Cancer Nurs.

[28] Ryan CJ, Zerwic JJ (2003). Perceptions of symptoms of myocardial infarction related to health care seeking behaviors in the elderly. J Cardiovasc Nurs.

[29] Elias T, Lowton K (2014). Do those over 80 years of age seek more or less medical help? A qualitative study of health and illness beliefs and behaviour of the oldest old. Sociol Health Illn.

[30] Kessler D, Lloyd K, Lewis G, Gray DP (1999). Cross sectional study of symptom attribution and recognition of depression and anxiety in primary care. BMJ.

[31] Coolbrandt A, Dierckx de Casterlé B, Wildiers H, Aertgeerts B, Van der Elst E, van Achterberg T, Milisen K (2016). Dealing with chemotherapy-related symptoms at home: a qualitative study in adult patients with cancer. Eur J Cancer Care (Engl).

[32] Rocha LS, Beuter M, Neves ET, Leite MT, Brondani CM, Perlini NMOG (2014). Self-care of elderly cancer patients undergoing outpatient treatment. Texto Contexto Enferm.

[33] Kidd L, Hubbard G, O'Carroll R, Kearney N (2009). Perceived control and involvement in self care in patients with colorectal cancer. J Clin Nurs.

[34] Loerzel VW, Clochesy J, Geddie P (2020). Using serious games to increase prevention and self-management of chemotherapy-induced nausea and vomiting in older adults with cancer. Oncol Nurs Forum.

[35] Cameron LD, Moss-Morris R, Kaptein A, Weinman J (2004). Illness-related cognition and behaviour. Health Psychology.

[36] Chao Y, Scherer YK, Wu Y, Lucke KT, Montgomery CA (2013). The feasibility of an intervention combining self-efficacy theory and Wii Fit exergames in assisted living residents: a pilot study. Geriatr Nurs.

[37] Maillot P, Perrot A, Hartley A (2012). Effects of interactive physical-activity video-game training on physical and cognitive function in older adults. Psychol Aging.

[38] Finkelstein J, Bedra M (2014). Avatar-based interactive diabetes education in older adults. J Am Geriatr Soc.

[39] Baranowski T, Buday R, Thompson DI, Baranowski J (2008). Playing for real: video games and stories for health-related behavior change. Am J Prev Med.

[40] Thompson D (2012). Designing serious video games for health behavior change: current status and future directions. J Diabetes Sci Technol.

[41] McCallum S (2012). Gamification and serious games for personalized health. Stud Health Technol Inform.

[42] Radhakrishnan K, Toprac P, O'Hair M, Bias R, Kim MT, Bradley P, Mackert M (2016). Interactive digital e-Health game for heart failure self-management: a feasibility study. Games Health J.

[43] Valladares-Rodriguez S, Fernández-Iglesias MJ, Anido-Rifón Luis, Facal D, Rivas-Costa C, Pérez-Rodríguez R (2019). Touchscreen games to detect cognitive impairment in senior adults. A user-interaction pilot study. Int J Med Inform.

[44] Binder JC, Zöllig J, Eschen A, Mérillat S, Röcke C, Schoch S, Jäncke L, Martin M (2015). Multi-domain training in healthy old age: Hotel Plastisse as an iPad-based serious game to systematically compare multi-domain and single-domain training. Front Aging Neurosci.

[45] Santos LH, Okamoto K, Hiragi S, Yamamoto G, Sugiyama O, Aoyama T, Kuroda T (2019). Pervasive game design to evaluate social interaction effects on levels of physical activity among older adults. J Rehabil Assist Technol Eng.

[46] Ingadottir B, Blondal K, Thue D, Zoega S, Thylen I, Jaarsma T (2017). Development, usability, and efficacy of a serious game to help patients learn about pain management after surgery: an evaluation study. JMIR Serious Games.

[47] Lunardini F, Borghese NA, Piccini L, Bernardelli G, Cesari M, Ferrante S (2020). Validity and usability of a smart ball-driven serious game to monitor grip strength in independent elderlies. Health Informatics J.

[48] Money AG, Atwal A, Boyce E, Gaber S, Windeatt S, Alexandrou K (2019). Falls Sensei: a serious 3D exploration game to enable the detection of extrinsic home fall hazards for older adults. BMC Med Inform Decis Mak.

[49] Kakulla BN 2020 tech trends of the 50+. AARP Research.

[50] Kakulla B (2021). Personal tech and the pandemic: older adults are upgrading for a better online experience. AARP Research.

[51] (2020). Antiemisis. National Comprehensive Cancer Network.

[52] Loerzel VW, Aroian K (2013). "A bump in the road": older women's views on surviving breast cancer. J Psychosoc Oncol.

[53] Loerzel VW, McNees P, Powel LL, Su X, Meneses K (2008). Quality of life in older women with early-stage breast cancer in the first year of survivorship. Oncol Nurs Forum.

[54] Geddie PI, Loerzel VW, Norris AE (2016). Family caregiver knowledge, patient illness characteristics, and unplanned hospital admissions in older adults with cancer. Oncol Nurs Forum.

[55] Donovan HS, Ward S, Sherwood P, Serlin RC (2008). Evaluation of the Symptom Representation Questionnaire (SRQ) for assessing cancer-related symptoms. J Pain Symptom Manage.

[56] Aaronson NK, Ahmedzai S, Bergman B, Bullinger M, Cull A, Duez NJ, Filiberti A, Flechtner H, Fleishman SB, de Haes J C (1993). The European Organization for Research and Treatment of Cancer QLQ-C30: a quality-of-life instrument for use in international clinical trials in oncology. J Natl Cancer Inst.

[57] Park S, Kim IR, Baek KK, Lee SJ, Chang WJ, Maeng CH, Hong JY, Choi MK, Kim YS, Sun JM, Ahn JS, Park K, Jo J, Jung SH, Ahn MJ (2013). Prospective analysis of quality of life in elderly patients treated with adjuvant chemotherapy for non-small-cell lung cancer. Ann Oncol.

[58] Scarpa M, Di Cristofaro L, Cortinovis M, Pinto E, Massa M, Alfieri R, Cagol M, Saadeh L, Costa A, Castoro C, Bassi N, Ruffolo C (2013). Minimally invasive surgery for colorectal cancer: quality of life and satisfaction with care in elderly patients. Surg Endosc.

[59] (2017). Technology use among seniors. Pew Research Center.

[60] Harris PA, Taylor R, Minor BL, Elliott V, Fernandez M, O'Neal L, McLeod L, Delacqua G, Delacqua F, Kirby J, Duda SN (2019). The REDCap consortium: building an international community of software platform partners. J Biomed Inform.

[61] Ballinger GA (2004). Using generalized estimating equations for longitudinal data analysis. Organ Res Methods.

[62] (2020). Research supported by NINR. National Institute of Nursing Research.

